# The heterometallic one-dimensional solvated coordination polymer [NiPt_2_Cl_6_(TRIP-Py)_4_]_*n*_

**DOI:** 10.1107/S2053229623001845

**Published:** 2023-03-09

**Authors:** Hans Gildenast, Lukas Gruszien, Ulli Englert

**Affiliations:** a Institute of Inorganic Chemistry, RWTH Aachen University, Landoltweg 1, 52074 Aachen, Germany; University of the Witwatersrand, South Africa

**Keywords:** heterometallic, coordination polymer, synchrotron, platinum, nickel, crystal structure, bypass algorithm, solvent mask, caged triptycene

## Abstract

The ditopic ligand 10-[4-(pyridin-4-yl)phen­yl]-9-phospha-10-silatriptycene (TRIP-Py, C_29_H_20_NPSi) binds as a pyridine donor to Ni^II^ and as a phosphatriptycene donor towards Pt^II^. The selectivity relies entirely on the Pearson character of the donor sites and the matching hardness of the respective metal cations. The product is the one-dimensional coordination polymer *catena*-poly[[[di­chlorido­nickel(II)]-bis­{μ-10-[4-(pyridin-4-yl)phen­yl]-9-phospha-10-silatrip­tycene}-bis­[di­chlorido­platinum(II)]-bis­{μ-10-[4-(pyridin-4-yl)phen­yl]-9-phospha-10-sila­trip­tycene}] di­chloro­methane penta­solvate ethanol icosa­solvate], {[NiPt_2_Cl_6_(TRIP-Py)_4_]·5CH_2_Cl_2_·20EtOH}_*n*_ (**1**), which retains large pores due to the in­herent rigidity of the ligand. This is enabled by the caged triptycene scaffold which fixes the direction of the phospho­rus donor with respect to the remaining mol­ecule and especially the pyridyl moiety. In its crystal structure, which was determined from synchrotron data, the pores of the polymer are filled with di­chloro­methane and ethanol mol­ecules. Finding a suitable model for the pore content is com­plicated as it is too disordered to give a reasonable atomic model but too ordered to be described by an electron gas solvent mask. This article presents an in-depth description of this polymer, as well as a discussion on the use of the bypass algorithm for solvent masks.

## Abstract 2

The heterofunctional ligand TRIP-Py cooordinates to Pt^II^ cations with its phosphatriptycene P-atom donor and to Ni^II^ cations with its pyridyl N-atom donor, giving a one-dimensional heterometallic coordination polymer.

## Introduction

The research area of coordination polymers (CPs) has become an established field in modern inorganic and coordination chemistry over recent decades (Batten *et al.*, 2008 ▸). CPs offer the possibility to adjust the material properties not just through the design of the ligand and the choice of the metal cation, but also through the dimensionality and topology of the CP. This allows a tailoring for a vast range of applications from catalysis, magnetism and optics to chemical separation, medicine and electrochemistry (Wang *et al.*, 2020 ▸; Zhong *et al.*, 2022 ▸; Yu *et al.*, 2022 ▸; Zhou *et al.*, 2022 ▸; Zhang *et al.*, 2021 ▸; Indra *et al.*, 2018 ▸). Controlling and understanding the properties of a CP requires information on its structure, making diffraction techniques indispensable for the field. As the growth of large single crystals of CPs can be quite challenging due to their inherent insolubility, the field profits heavily from high-flux X-ray sources like synchrotron facilities and modern techniques like electron diffraction (Balestri *et al.*, 2019 ▸; Huang *et al.*, 2021 ▸).

While the vast majority of CPs contains a single type of metal cation, inter­est in heterometallic CPs containing two or more different metal cations in an orderly fashion is steadily growing (Kremer & Englert, 2018 ▸; Kuwamura & Konno, 2021 ▸). This inherently increases the synthetic challenge but opens an even larger playground to tune and combine properties. Gaining control over the position of the two different cations is frequently achieved by using heterofunctional ligands with distinctly different coordination sites. These can, for example, differ in their Pearson hardness (Pearson, 1963 ▸) and preferably coordinate metal cations of matching Pearson character.

**Figure 1:**
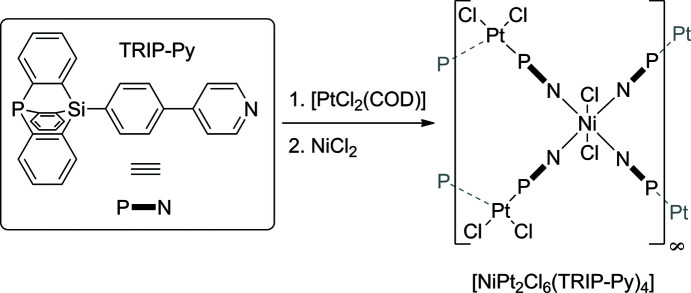
Reaction scheme for the synthesis of [NiPt_2_Cl_6_(TRIP-Py)_4_]_*n*_ and a simplified structural formula of the product.

In this article, we address the selectivity of a soft phos­pho­rus and a harder nitro­gen donor. This combination has been demonstrated to give selective heterometallic coordination com­pounds for a long list of discrete metal com­plexes (Hara *et al.*, 2021 ▸; Schroers *et al.*, 2021 ▸). In CP chemistry, however, the same pair of donor sites has only very recently been used for a heterometallic CP (Gildenast *et al.*, 2022*a* ▸). The ligand used in this previous report on heterometallic Zn^II^/Hg^II^ polymers and also in the construction of the title com­pound is a rigid linear linker combining a pyridyl moiety with a phosphatriptycene, abbreviated as TRIP-Py (Fig. 1 ▸).

The phosphatriptycene belongs to the family of caged phosphines and has unique properties due to its special geometry (Shet *et al.*, 2021 ▸; Tsuji *et al.*, 2006 ▸). The introduction of the secondary bridgehead forces the phenyl­ene propellers to be parallel to the phospho­rus lone pair. Thus, the H atoms are pointing in the same direction increasing steric demand. Accordingly, until our recent publication (Gildenast *et al.*, 2022*b* ▸), no metal com­plex with more than two phosphatriptycene ligands bound to a single metal cation had been reported. At the same time, the geometry forces acute C—P—C angles which increases the *s*-character of the lone pair, lowering its basicity and σ-donor strength while increasing the π acidity (Agou *et al.*, 2004 ▸; Freijee & Stam, 1980 ▸; Jongsma *et al.*, 1974 ▸; Drover *et al.*, 2018 ▸; Hu *et al.*, 2019 ▸; Mahaut *et al.*, 2022 ▸). This strengthens the bond, especially towards electron-rich metal cations (Cao *et al.*, 2019 ▸; Hu *et al.*, 2021 ▸).

In this article, we present the crystallization and particularly challenging structural investigation of a desolvation-labile heterometallic CP in which TRIP-Py connects the softer Pt^II^ and the harder Ni^II^ cations. In contrast to our previously reported structures involving TRIP-Py, the halides coordinated at either metal cation are not engaged in polymer expansion and remain strictly terminal.

## Experimental

**Figure 2:**
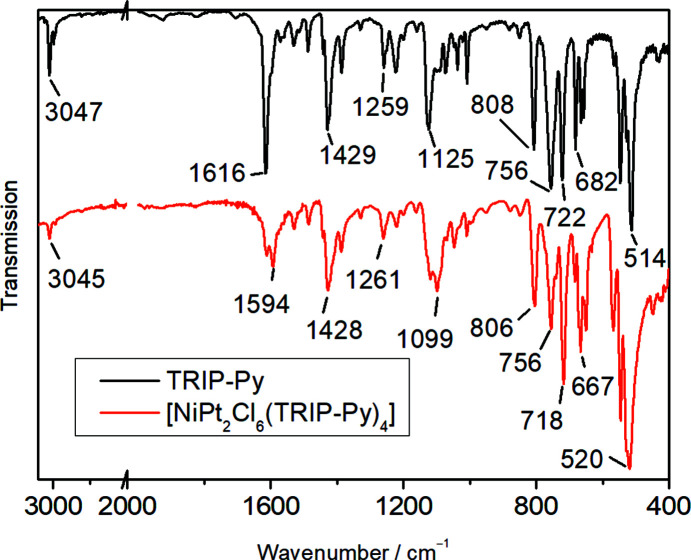
Comparison of the IR spectra of uncoordinated TRIP-Py and [NiPt_2_Cl_6_(TRIP-Py)_4_]_*n*_. The wavenumber axis is stretched between 2000 and 400 cm^−1^.

Unless stated otherwise, all reagents and solvents were obtained from commercial sources and used without further purification. TRIP-Py and [PtCl_2_(COD)] were prepared according to published procedures (Gildenast *et al.*, 2022*a* ▸; Brauer, 1981 ▸). For the single-crystal X-ray diffraction measurement, the κ goniometer at PETRA-III, P24, EH1, was used. The instrument was equipped with a Dectris CdTe area detector. For our experiment, synchrotron radiation (25 keV, λ = 0.500 Å) was used at a temperature of 100 (2) K (Oxford Cryostream 600 instrument, Oxfordshire, UK). Data were integrated with *XDS* (Kabsch, 2010 ▸) and corrected for absorption by multi-scan methods with *SADABS* (Bruker, 2014 ▸). The powder diffraction patterns were recorded at the Institute of Inorganic Chemistry, RWTH Aachen University, using a curved Stoe imaging-plate detector (IP-PSD). The diffractogram was recorded on a flat sample at ambient temperature in transmission using Cu *K*α_1_ radiation. The ATR FT–IR spectrum was measured with a Shimadzu IRSpirit with a QATR-S ATR unit equipped with a diamond prism and is shown in Fig. 2 ▸. It immediately shows the presence of the ditopic ligand in the solid. In the range between 1600 and 500 cm^−1^, the spectrum reflects the pattern observed for uncoordinated TRIP-Py (Gildenast *et al.*, 2022*a* ▸). The ele­men­tal analysis (CHN) was measured using a HERAEUS CHNO-Rapid VarioEL. The thermogravimetric (TGA) measurements were carried out with a Netzsch STA 409 C/CD in a flux of air (60 ml min^−1^) at a heating rate of 5 K min^−1^ on a sample dried in air. The EDX measurement was performed in a Leo/ZeissFE-SEM Supra 35 VP instrument equipped with an OxfordINCA Energy 200 (SiLi crystal, 133 eV, 10 mm^2^).

### Synthesis and crystallization

TRIP-Py (17.6 mg, 0.04 mmol) and [PtCl_2_(COD)] (7.5 mg, 0.02 mmol) were each dissolved in di­chloro­methane (1 ml each) and the solutions were combined. NiCl_2_·6H_2_O (2.4 mg, 0.01 mmol) was dissolved in ethanol (1 ml). The two solutions were layered with a layer of the mixed solvents (1 ml) in between. After several days, light-green crystals of **1** were obtained. For bulk analyses, they were isolated by filtration and washed with ethanol (yield: 14.6 mg, 60%).

### Refinement

**Figure 3:**
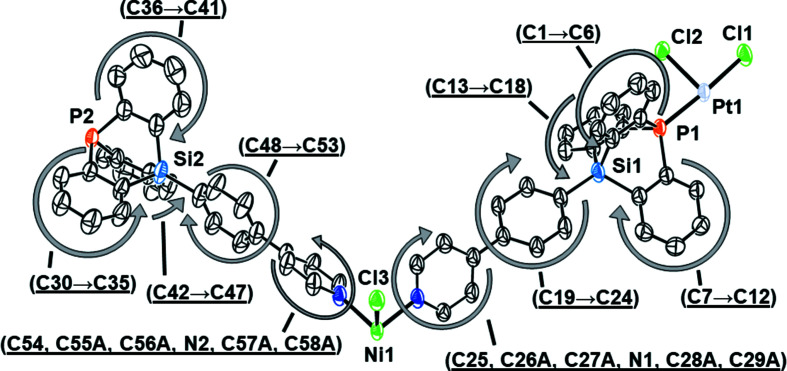
Displacement ellipsoid plot of the asymmetric unit of [NiPt_2_Cl_6_(TRIP-Py)_4_]_*n*_ in **1** (40% probability level), with labels for the atom sites. H atoms and alternative conformations for the disordered pyridyl rings have been omitted for clarity.

Crystal data, data collection and structure refinement details for **1** are summarized in Table 1 ▸ and the asymmetric unit is shown in Fig. 3 ▸.

H atoms attached to C atoms were introduced in calculated positions and treated as riding, with *U*_iso_(H) = 1.2*U*_eq_(C). For the pyridyl rings, split positions were refined for the C atoms in positions 2, 3, 5 and 6 with respect to the nitro­gen. Only a site occupancy of 0.5 is com­patible with reasonable inter­atomic distances between neighbouring pyridyl rings. The contribution of pore-contained solvent to the structure factors was treated with the bypass algorithm as implemented in SQUEEZE in *PLATON* (van der Sluis & Spek, 1990 ▸; Spek, 2015 ▸); a detailed discussion of alternative approaches is given in Section 3 (*Results and discussion*).

## Results and discussion

**Figure 4:**
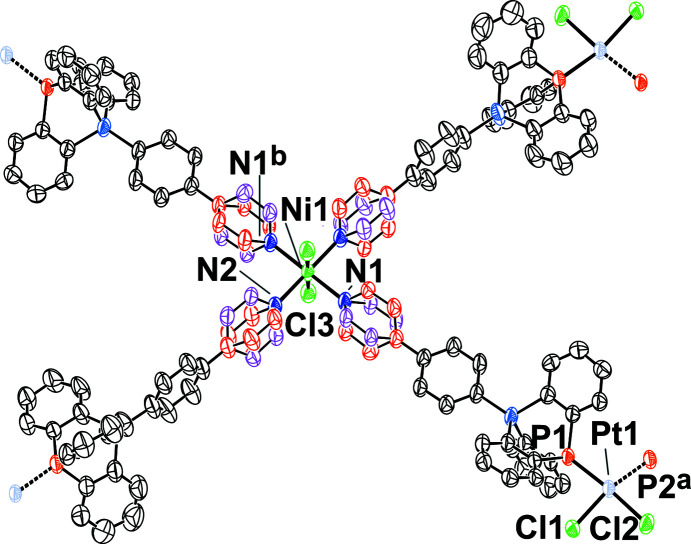
Displacement ellipsoid plot of [NiPt_2_Cl_6_(TRIP-Py)_4_]_*n*_ in **1** (50% probability level, space group *P* 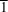 , *Z* = 1) prepared with *PLATON* (Spek, 2020 ▸). The two concerted local conformations for the pyridyl rings are coloured red and pink. Selected inter­atomic distances and angles (Å, °): Ni1—N1 2.100 (4), Ni1—N2 2.107 (3), Ni1—Cl3 2.4467 (18), τ_4_(Pt1) = 0.19 and Var(*X*—Pt1—*Y*) = 1650.9°^2^ (Yang *et al.*, 2007 ▸). [Symmetry codes: (*a*) *x* − 1, *y*, *z* − 1; (*b*) −*x* + 1, −*y* + 2, −*z* + 1.]

The title com­pound, [NiPt_2_Cl_6_(TRIP-Py)_4_]_*n*_, was prepared by reactive diffusion crystallization of an *in-situ*-generated di­chloro­methane solution of the com­plex [PtCl_2_(TRIP-Py)_2_] with an ethano­lic solution of NiCl_2_. The insoluble product is a heterometallic coordination polymer connected *via* covalent and coordinative bonds in one spatial direction (Fig. 4 ▸).

**Figure 5:**
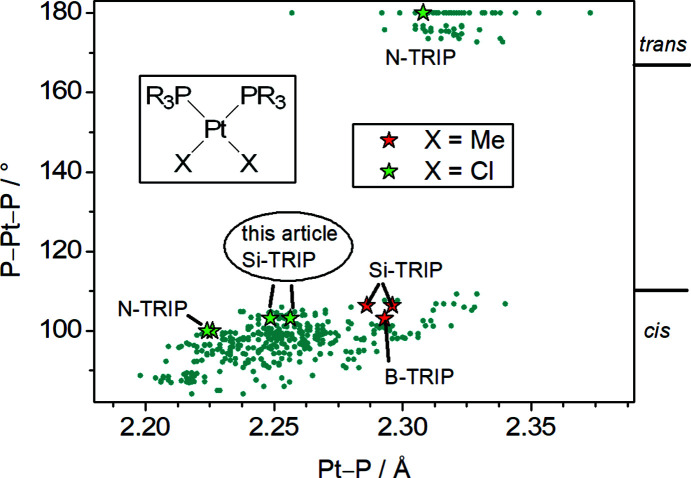
Scatter plot for the geometry of [Pt*X*_2_(P*R*_3_)_2_] com­plexes (*X* = halide or methyl and P*R*_3_ = tertiary phosphine). The P—Pt—P angle is plotted against the Pt—P distance. The data for the shown fragment were extracted from the CSD (Groom *et al.*, 2016 ▸). The search was limited to error-free data sets collected at *T* ≤ 200 K with *R*_1_ ≤ 0.05. Polymers and disordered structures were excluded, as well as structures of chelating phosphines with a C_2_ bridge. All Pt com­plexes of phosphatriptycenes are added to the plot with star-shaped data points, and their respective secondary heteroatom is noted as *Y*-TRIP, with *Y* being either B, N or Si. Additionally, the data from the structure presented in this article are noted with *this article*. The colours of the stars denote whether they are chloride or methyl com­plexes (Drover *et al.*, 2018 ▸; Tsuji *et al.*, 2006 ▸; Ube *et al.*, 2017 ▸).

The Ni^II^ cation is located on a crystallographic centre of inversion and resides in pseudo-octa­hedral coordination by two chloride ligands and four pyridyl donors of TRIP-Py ligands. Steric repulsion between *ortho* H atoms of adjacent ligands and between pyridyl H atoms and the halide ligands requires a tilt of the heteroaromatic rings. As a continuous windmill arrangement is incom­patible with the inversion symmetry, disorder with alternative ring conformations of exactly half site occupancy is enforced. Each [NiCl_2_(TRIP-Py)_4_] cross is connected to the next one *via* two PtCl_2_ moieties with the P-atom donors in a *cis* configuration, resulting in a one-dimensional CP along [101]. Fig. 5 ▸ shows a scatter plot for the geometry of [Pt*X*_2_(P*R*_3_)_2_] com­plexes and clearly displays the expected binodal distribution of the Pt—P distances, with the *trans* com­plexes showing systematically larger values as two π acceptors are opposed and com­pete for backbonding from the same metal *d* orbital. The data for the examples with phosphatriptycenes are especially highlighted, including the data from this article.

The plot shows that the Pt—P distances for phosphatriptycenes are very com­parable to those of regular uncaged phosphines. In contrast, the metal–ligand distances in Au^I^ com­plexes of phosphatriptycenes (Gildenast *et al.*, 2022*a* ▸) are among the shortest of all phosphines in the Cambridge Structural Database (CSD; Version 5.43, with updates from November 2022; Groom *et al.*, 2016 ▸). We speculate that π backbonding may play a less pronounced role in the case of the Pt^II^ cation with its more positive formal charge. The P—Pt—P angle, however, is systematically at the larger end of the spectrum for phosphatriptycenes. The repulsion of the large triptycene moieties distorts the coordination sphere around the Pt^II^ cation increasing the P—Pt—P angle and com­pressing the three remaining *cis* angles. Additionally, a reduction in planarity of the coordination sphere occurs com­pared to the *cis*-PtCl_2_ com­plex of the uncaged phosphine PPh_3_ (Table 2 ▸).

**Figure 6:**
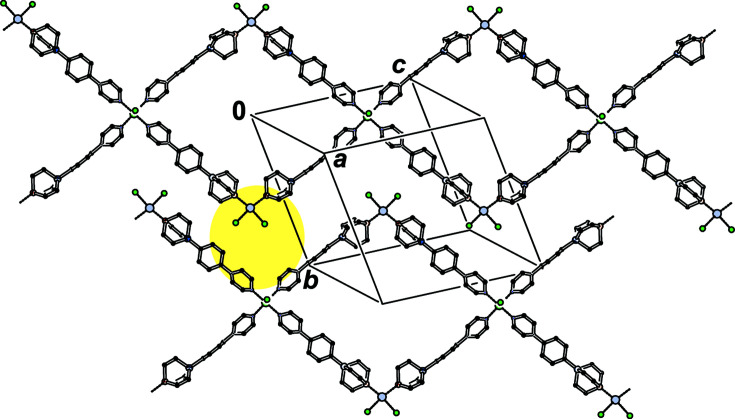
Packing of two neighbouring polymer strands of [NiPt_2_Cl_6_(TRIP-Py)_4_]_*n*_ in **1** shown along [311]. H atoms have been omitted and the triptycene wings simplified for clarity. The yellow ellipse shows how adjacent polymers are inter­digitated.

There are very few contacts between individual polymer strands close to the sum of their van der Waals radii. This includes a contact between an aromatic H atom and a chloride ligand attached to the Ni centre [Cl3⋯H4^*a*^ = 2.92 Å; symmetry code: (*a*) −*x*, −*y* + 2, −*z* + 1], an aromatic H atom pointing towards the centre of an aromatic ring [C10⋯H39^*b*^ = 2.83 Å; symmetry code: (*b*) *x*, *y*, *z* − 1] and two aromatic C atoms around an inversion centre which puts them in a potential π-stacking position [C45⋯C45^*c*^ = 3.369 (9) Å; symmetry code: (*c*) −*x* + 2, −*y* + 1, −*z* + 2]. In none of these does the mol­ecular arrangement suggest a strong inter­action. Instead, there is a distinct packing feature with the PtCl_2_ corner of each [Ni_2_Pt_2_(TRIP-Py)_4_] parallelogram pointing roughly towards the re-entrant corner of the NiCl_2_ vertex of a neighbouring strand. This results in a presumably weak offset π-stacking inter­action [C16⋯C18^*d*^ = 3.620 (5) Å; symmetry code: (*d*) −*x*, −*y* + 1, −*z* + 1]. Fig. 6 ▸ shows how adjacent polymers are inter­digitated.

**Figure 7:**
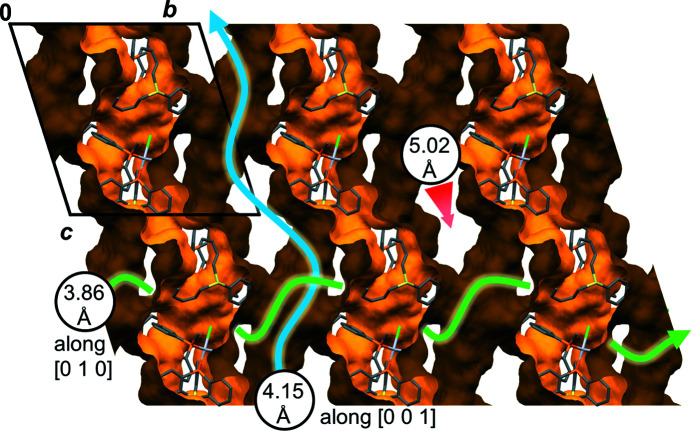
Packing of 1 × 3 × 2 unit cells of **1** with a void contact surface calculated with *Mercury* (Macrae *et al.*, 2020 ▸) (1.2 Å probe radius, 0.3 Å grid spacing). The spheres represent the diameter of the largest possible sphere that can pass through the pore along the given unit-cell vectors (Willems *et al.*, 2012 ▸).

The centre of the parallelogram also corresponds to the largest pore along [100] (Fig. 7 ▸). The diameter of the largest possible sphere that can pass through this pore has been determined with *Zeo++* (Willems *et al.*, 2012 ▸) and amounts to 5.02 Å. The pores along [010] and [001] are slightly more narrow with limiting diameters of 3.86 and 4.15 Å, respectively, and have much more contorted pathways. Depending on which size is used for the probe radius, the three-dimensional pore system com­prises between 52 and 56% of the unit-cell volume (SQUEEZE in *PLATON*, 1.5 and 1.0 Å probe radius, electron count remains roughly the same, <4% discrepancy).

**Figure 8:**
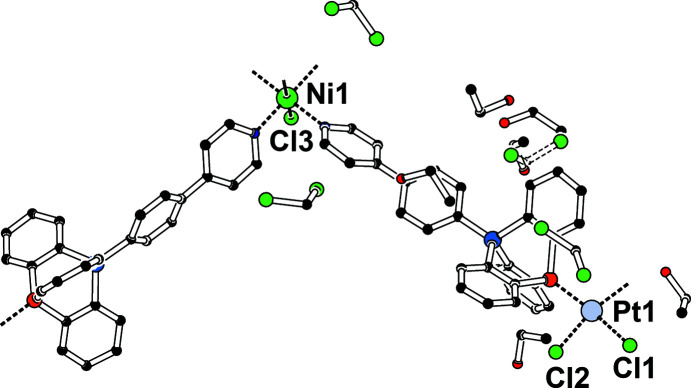
The asymmetric unit of **1**, with a partial mol­ecular model of the solvent-filled pore. H atoms have been omited for clarity. The di­chloro­methane mol­ecule shown as dashed is only partially occupied and overlaps with the position of the adjacent ethanol mol­ecule.

The pore contains strongly disordered solvent mol­ecules. Based on preliminary distances between residual electron-density peaks and in agreement with the solvents employed in the synthesis, both di­chloro­methane and ethanol mol­ecules are present. A tentative refinement of solvent mol­ecules was performed, and 5.6 di­chloro­methane and 10.8 ethanol mol­ecules per unit cell could be assigned in this model **A** (Fig. 8 ▸).

**Figure 9:**
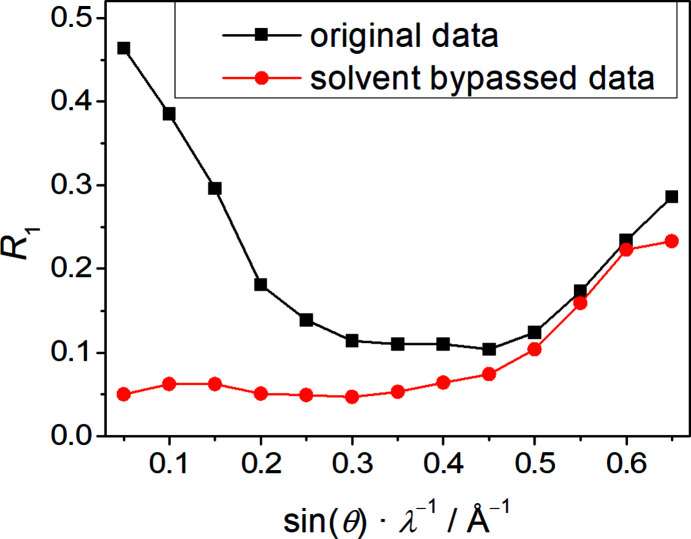
Plot for **1** of the agreement factor *R*_1_ against the diffraction resolution for the original data and the data modified with the bypass algorithm as implemented in *PLATON* (van der Sluis & Spek, 1990 ▸; Spek, 2015 ▸).

On the one hand, the above-mentioned solvent model **A** is not fully satisfactory: it did not account for the com­plete pore space but left a discrete void and a thin solvent-accessible channel, with a combined volume of 871 Å per unit cell. Despite the combined use of rigid fragments and hard geometry restraints for the solvent part, this partial solvent model **A** did not converge without damping, most probably because of high correlation between refinement variables describing the solvent. On the other hand, the graphical synopsis of the solvent-masking process in Fig. 9 ▸ indicates that ‘squeezing out’ the entire solvent-filled pore according to model **B** is an equally crude approximation.

Fig. 9 ▸ shows that the contribution of the solvent mol­ecules to the structure factors extends up to a resolution of 0.4 Å^−1^, *i.e.* almost into atomic resolution. The solvent part is at least in part associated with long-range order and cannot be well modelled by an electron gas. This explains why the solvent-squeezed structure model **B** retains a significant number of disagreeable intensities in the inter­mediate resolution range. These unsatisfactory intensity data show better agreement with the calculated structure factors from the partial solvent model **A**. In conclusion, we decided to report the more straightforward model **B** because localization of individual solvent mol­ecules is not a crucial feature for the title structure. The overall content of the pore can be estimated from the results of the bypass algorithm as summarized in Table 3 ▸.

For the estimation of the spatial demand of a disordered solvent mol­ecule, we followed the suggestion of Mecozzi & Rebek (1998 ▸) and assumed a 1.3-fold of the volume of the mol­ecules in their own crystal structure. A combination of 5 di­chloro­methane (DCM) and 20 ethanol mol­ecules per unit cell represents a good fit to pore volume and electron count. This amount of di­chloro­methane mol­ecules is slightly lower than in our tentative mol­ecular solvent model **A**, but we recall that this model is rather unstable. The large number of volatile solvent mol­ecules also makes the com­pound prone to rapid desolvation. This impairs a reasonable validation of the structure by powder diffraction. We measured powder pat­terns of both wet crystals taken directly from the mother liquor, as well as dried samples. Both of them look quite similar but do not match the phase characterized by single crystal X-ray diffraction. The loss of solvent mol­ecules is also reflected in the elemental analysis which matches more closely the expected values of the desolvated polymer with small amounts of residual solvent (Table 4 ▸). The best match for the experimentally determined values is achieved for two di­chloro­methane mol­ecules per unit cell.

**Figure 10:**
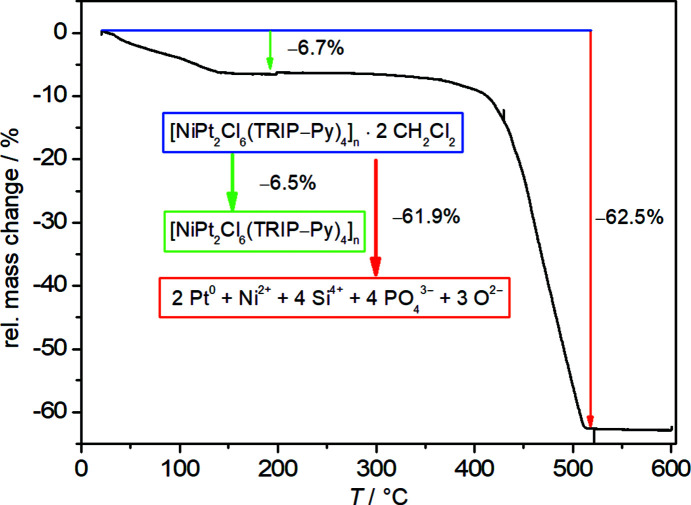
Thermogravimetric analysis of [NiPt_2_Cl_6_(TRIP-Py)_4_]_*n*_, with a heating rate of 5 K min^−1^ in a stream of air.

The elemental analysis matches the results obtained in the thermogravimetric analysis (Fig. 10 ▸). {[NiPt_2_Cl_6_(TRIP-Py)_4_]·2DCM}_*n*_ loses weight in two well-separated steps. First, a gradual loss of 6.7% of mass until 160 °C is observed, which agrees with the desolvation of two di­chloro­methane mol­ecules. The second step begins at 350 °C and ends at 520 °C, after which 37.5% of the original sample weight is left. The identity of the remaining black powder could not be identified unambiguously. Its diffraction pattern displays merely reflections for elemental Pt. These are very broad, indicating a small average particle size. Using energy-dispersive X-ray spectroscopy (EDX), the elements Pt, Ni, Si and P were detected in a ratio of 2.0 (3):1.3 (4):4.1 (5):4.0 (5), in acceptable match with the com­position in the original CP. From this we propose that Ni and Si stay in their oxidation states Ni^II^ and Si^IV^, that phos­pho­rus is oxidized to phosphate anions and oxide anions balance the remaining positive charge. The total sum formula of this mixture has a mol­ecular weight of 38.1% of the original CP and two mol­ecules of di­chloro­methane, matching the experimental weight loss from the TGA measurement. The EDX measurement does however reveal a higher than ex­pected value for oxygen. Our suggested com­position would require a Pt:O ratio of 2:19; the EDX analysis yields 2.0 (3):34 (3). This discrepancy may be caused by a contribution to the oxygen signal from the material used for fixing the sample.

## Conclusion

The structural characterization of **1** proved challenging but also rewarding. The PtCl_2_ moieties in the heterometallic polymer are exposed towards the periphery and therefore potentially useful for follow-up reactions. They might, for example, represent analytically active sites which could be tested in future experiments. The unique electronic properties of the phosphatriptycene can lead to inter­esting reactivities, and the very low solubility of the coordination polymer enables a simple separation of the catalyst from potential products.

## Supplementary Material

Crystal structure: contains datablock(s) I, global. DOI: 10.1107/S2053229623001845/ef3041sup1.cif

Structure factors: contains datablock(s) I. DOI: 10.1107/S2053229623001845/ef3041Isup2.hkl

CCDC reference: 2245178

## Figures and Tables

**Table 1:** Experimental details

Crystal data	
Chemical formula	[NiPt_2_Cl_6_(C_29_H_20_NPSi)_4_]·5CH_2_Cl_2_·20C_2_H_6_O
*M*_r_	3773.65
Crystal system, space group	Triclinic, *P* 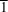
Temperature (K)	100
*a*, *b*, *c* (Å)	12.702 (7), 19.372 (10), 20.340 (7)
α, β, γ (°)	71.313 (7), 81.809 (13), 78.917 (19)
*V* (Å^3^)	4635 (4)
*Z*	1
Radiation type	Synchrotron, λ = 0.500 Å
μ (mm^−1^)	0.78
Crystal size (mm)	0.20 × 0.20 × 0.10

Data collection	
Diffractometer	Area-detector Dectris CdTe on a κ goniometer at EH1 P24, DESY
Absorption correction	Multi-scan (*SADABS*; Bruker, 2014 ▸)
*T*_min_, *T*_max_	0.775, 0.837
No. of measured, independent and observed [*I* > 2σ(*I*)] reflections	161591, 20869, 16117
*R*_int_	0.063
(sin θ/λ)_max_ (Å^−1^)	0.657

Refinement	
*R*[*F* ^2^ > 2σ(*F* ^2^)], *wR*(*F* ^2^), *S*	0.036, 0.108, 1.03
No. of reflections	20869
No. of parameters	691
H-atom treatment	H-atom parameters constrained
Δρ_max_, Δρ_min_ (e Å^−3^)	1.58, −1.04

Computer programs: *KAPPA* (Paulmann, 2023 ▸), *XDS2022* (Kabsch, 2010 ▸), *SHELXT2018* (Sheldrick, 2015*a* ▸), *SHELXL2018* (Sheldrick, 2015*b* ▸) and *PLATON* (Spek, 2020 ▸).

**Table 2:** Selection of geometrical parameters of the Pt^II^ coordination sphere of **1** and two solvates of [PtCl_2_(PPh_3_)_2_] (Miao *et al.*, 2009 ▸; Al-Fawaz *et al.*, 2004 ▸) representing the uncaged phosphines; discrepancies from planarity can be detected using the τ_4_ parameter (Yang *et al.*, 2007 ▸) and the dihedral angle *φ* between Pt1/P1/P2 and Pt1/Cl1/Cl2

	[NiPt_2_Cl_6_(TRIP-Py)_4_]·5CH_2_Cl_2_·20EtOH	[PtCl_2_(PPh_3_)_2_]·CHCl_3_	[PtCl_2_(PPh_3_)_2_]·3CHCl_3_
Pt1—P1	2.2486 (17)	2.2481 (18)	2.2560 (19)
Pt1—P2	2.2563 (16)	2.266 (2)	2.2708 (19)
Pt1—Cl1	2.3428 (19)	2.324 (2)	2.353 (2)
Pt1—Cl2	2.3337 (17)	2.3548 (19)	2.350 (2)
P1—Pt1—P2	103.09 (5)	97.43 (7)	98.74 (7)
Cl1—Pt1—Cl2	86.88 (4)	86.48 (7)	85.24 (7)
P1—Pt1—Cl2	87.01 (4)	89.85 (7)	91.01 (7)
P2—Pt1—Cl1	84.85 (4)	86.26 (7)	85.11 (7)
τ_4_	0.19	0.08	0.10
φ(PtCl_2_, PtP_2_)	14.04 (6)	2.01 (10)	3.69 (10)

**Table 3:** Void volume (*V*) and electron content (e^−^) according to the program SQUEEZE (Spek, 2015 ▸) as implemented in *PLATON* (Spek, 2020 ▸), and the average electron count per volume in the void ρ for the pore treated with the bypass algorithm The values for *V* for di­chloro­methane and ethanol were taken from their crystal structures (Kawaguchi *et al.*, 1973 ▸; Jönsson, 1976 ▸) and multiplied by 1.3 (Mecozzi & Rebek, 1998 ▸).

	*V* (Å^3^)	e^−^	ρ (e^−^ Å^−3^)
Void	2528	740	0.29
CH_2_Cl_2_	107	42	0.39
EtOH	97	26	0.27
5CH_2_Cl_2_ + 20EtOH	2475	730	0.29

**Table 4:** CHN elemental analysis (%) for the bulk material of [NiPt_2_Cl_6_(TRIP-Py)_4_]_*n*_ (the sample was prepared by drying in air)

	C	H	N
Analysis calculated for desolvated (C_116_H_80_Cl_6_N_4_NiP_4_Pt_2_Si_4_)	57.39	3.32	2.31
Analysis calculated for ·5DCM·20EtOH (C_161_H_210_Cl_16_N_4_NiO_20_P_4_Pt_2_Si_4_)	51.24	5.61	1.48
Analysis calculated for ·2DCM (C_118_H_84_Cl_10_N_4_NiP_4_Pt_2_Si_4_)	54.56	3.26	2.16
Found	53.98	3.37	2.15
